# GroundsWell: Community-engaged and data-informed systems transformation of Urban Green and Blue Space for population health – a new initiative

**DOI:** 10.12688/wellcomeopenres.18175.1

**Published:** 2022-09-20

**Authors:** Ruth F. Hunter, Sarah E. Rodgers, Jeremy Hilton, Mike Clarke, Leandro Garcia, Catharine Ward Thompson, Rebecca Geary, Mark A. Green, Ciaran O'Neill, Alberto Longo, Rebecca Lovell, Alex Nurse, Benedict W. Wheeler, Sarah Clement, Ana Porroche-Escudero, Rich Mitchell, Ben Barr, John Barry, Sarah Bell, Dominic Bryan, Iain Buchan, Olly Butters, Tom Clemens, Natalie Clewley, Rhiannon Corcoran, Lewis Elliott, Geraint Ellis, Cornelia Guell, Anna Jurek-Loughrey, Frank Kee, Aideen Maguire, Simon Maskell, Brendan Murtagh, Grahame Smith, Timothy Taylor, Ruth Jepson, GroundsWell Consortium

**Affiliations:** 1 Centre for Public Health, Queen's University Belfast, Belfast, UK; 2 Department of Public Health, Policy & Systems, University of Liverpool, Liverpool, UK; 3 School of Defence and Security, Cranfield University, Bedfordshire, UK; 4 OPENspace research centre, University of Edinburgh, Edinburgh, UK; 5 Department of Geography & Planning, University of Liverpool, Liverpool, UK; 6 School of Biological Sciences, Queen's University Belfast, Belfast, UK; 7 European Centre for Environment and Human Health, University of Exeter Medical School, Truro, UK; 8 Department of Geography and Planning, University of Western Australia, Perth, Australia; 9 Lancaster Environment Centre, Lancaster University, Lancaster, UK; 10 MRC/CSO Social and Public Health Sciences Unit, University of Glasgow, Glasgow, UK; 11 School of History, Anthropology, Philosophy and Politics, Queen's University Belfast, Belfast, UK; 12 School of Geosciences, University of Edinburgh, Edinburgh, UK; 13 Primary Care and Mental Health, University of Liverpool, Liverpool, UK; 14 School of Natural and Built Environment, Queen's University Belfast, Belfast, UK; 15 School of Electronics, Electrical Engineering and Computer Science, Queen's University Belfast, Belfast, UK; 16 Electrical Engineering and Electronics, University of Liverpool, Liverpool, UK; 17 Nursing and Allied Health, Liverpool John Moores University, Liverpool, UK; 18 Scottish Collaboration for Public Health Research and Policy (SCPHRP), University of Edinburgh, Edinburgh, UK

**Keywords:** Public health; non-communicable disease; green and blue space; complex systems; data science; citizen science; interdisciplinary; health inequalities

## Abstract

Natural environments, such as parks, woodlands and lakes, have positive impacts on health and wellbeing. Urban Green and Blue Spaces (UGBS), and the activities that take place in them, can significantly influence the health outcomes of all communities, and reduce health inequalities. Improving access and quality of UGBS needs understanding of the range of systems (e.g. planning, transport, environment, community) in which UGBS are located. UGBS offers an ideal exemplar for testing systems innovations as it reflects place-based and *whole society *processes *,* with potential to reduce non-communicable disease (NCD) risk and associated social inequalities in health. UGBS can impact multiple behavioural and environmental aetiological pathways. However, the systems which desire, design, develop, and deliver UGBS are fragmented and siloed, with ineffective mechanisms for data generation, knowledge exchange and mobilisation. Further, UGBS need to be co-designed with and by those whose health could benefit most from them, so they are appropriate, accessible, valued and used well.

This paper describes a major new prevention research programme and partnership, *GroundsWell*, which aims to transform UGBS-related systems by improving how we plan, design, evaluate and manage UGBS so that it benefits all communities, especially those who are in poorest health. We use a broad definition of health to include physical, mental, social wellbeing and quality of life. Our objectives are to transform systems so that UGBS are planned, developed, implemented, maintained and evaluated with our communities and data systems to enhance health and reduce inequalities.

GroundsWell will use interdisciplinary, problem-solving approaches to accelerate and optimise community collaborations among citizens, users, implementers, policymakers and researchers to impact research, policy, practice and active citizenship. GroundsWell will be shaped and developed in three pioneer cities (Belfast, Edinburgh, Liverpool) and their regional contexts, with embedded translational mechanisms to ensure that outputs and impact have UK-wide and international application.

## Disclaimer

The views expressed in this article are those of the author(s). Publication in Wellcome Open Research does not imply endorsement by Wellcome.

## Background

Several meta-analyses have demonstrated that Urban Green and Blue Spaces (UGBS) can have direct and indirect effects on non-communicable disease (NCD), summarising the strength of associations between greenspace exposure and reduced premature mortality, improved mental wellbeing and physical activity, and reduced inequalities in health ^1– 4^. UGBS also contribute to preventative health through wider co-benefits such as mitigating the effects of climate change, reducing the heat island effect and alleviating flood risk ^5– 16^. The provision of UGBS, and their benefits, are not equal. UGBS in low income areas are typically less extensive, poorer quality, and inaccessible, limiting their health benefits ^17, 18^. Further, many communities (i.e. social groups in deprived areas), who often have higher rates of NCD, tend not to be able or willing to access high quality UGBS. Such income related inequalities translate into inequalities in use that increased under COVID-19 restrictions in the UK ^19^.

The 2019 Lancet Obesity commission placed community engagement with systems science at the centre of the critical agenda to address both NCD and climate change ^20^. Systems science recognises that we all live and operate within a set of complex systems that interact and impact on any new or existing programme of work. Previous studies have successfully employed systems-based approaches (i.e. methods based on the structure and function of how systems operate and interact with each other) for community involvement ^21– 24^, and there is evidence that these approaches impact both community capacity building and intervention sustainability ^21, 24, 25^. Although there are significant challenges in undertaking high quality participatory research and decision-making, there is compelling evidence that doing so leads to more successful and sustainable change ^21^. However, these studies are the exception rather than the rule and have yet to be tested in UGBS.

UGBS are often viewed as discrete physical ‘assets’ in the planning process without adequate appreciation of their health benefits, or the accompanying social, environmental, economic and planetary health co-benefits. There is a lack of understanding of how: (1) UGBS are integrated within the surrounding urban fabric, connecting cultural and physical assets in the environment, and (2) how UGBS management and investment can support UGBS potential to enhance the social environment ^26^. UGBS are often developed with a focus on infrastructure and maintenance rather than community usage. This has led to criticisms that inequitable distribution of quality UGBS widen health and social inequalities through: inappropriate models of provision; degraded and devalued spaces; tension and contested priorities between diverse users of the space; and issues such as gentrification and degradation by tourism ^13^. While studies have assessed the value of UGBS, most have failed to capture *all* the health effects ^26^, and rarely consider the value of the wider public health and planetary health co-benefits of UGBS, such as employment, climate change mitigation ^27^. There is a need to consider the widest range of health and economic impacts, and what stakeholders value (and provide credible evidence for them), as this may alter prioritisation decisions ^28^. Evaluation of small scale projects is mainly piecemeal and rarely focusing on outcomes. As a result, the evidence base for promising projects is often undocumented, discounted or undervalued.

There is a need for a systems transformation in how policy, implementation, research, data providers (health, environment, social, economic), and communities work together, in order to realise the full benefits of UGBS. We will develop the basis for sustained systemic change in UGBS for: i) embedding communities into research, policy and practice; ii) creating, managing, analysing and sharing data; and iii) generating system-based solutions that benefit the population across the social gradient and all stakeholders. Learning from successful local solutions will inform systems-level transformations and allow for generalisable application.

In 2019, the UK Prevention Research Partnership (UKPRP) launched a second call of their novel model of public health funding to support research into the primary prevention of NCDs. Their aim is to develop innovative and interdisciplinary approaches and deliver upstream interventions to improve population health and reduce health inequalities. We have been awarded funding for 5-year years to develop the GroundsWell consortium.

## Aims and objectives

GroundsWell aims to co-produce a whole systems approach to UGBS to improve population health and reduce health inequalities. The main objective is to drive community innovation applying systems science that maximises the contribution of UGBS to the primary prevention of, and reduction of inequalities in, NCD in urban settings by addressing socio-economic causes ^29^.

The overarching research question is: *How can we optimise innovative systems-based transformations to UGBS to benefit communities at high risk of NCD?*

Our research will be conducted across seven interrelated and interacting Work Packages (WPs) each addressing specific research questions:

1. How do UGBS systems impact on public health and other broader co-benefits? How can these systems be transformed to support future solutions to prevent NCD and reduce inequalities in health? (WP1+2);
2. How can we ensure that the development, implementation and evaluation of UGBS interventions is community and systems focussed to prevent NCD and reduce inequalities? (WP3);
3. How would a dynamic data repository aid the development and evaluation of UGBS systems-wide interventions? (WP4);
4. What are the economic costs and benefits of UGBS for NCD prevention and inequality reduction? (WP5);
5. What is needed to ensure political and decision-making contexts and systems support and sustain UGBS policies for health? (WP6);
6. What are the most effective methods to ensure the impact of GroundsWell in supporting broader efforts to improve population health, NCD prevention and reduce inequalities? (WP7).

## Logic model

**Figure 1.**
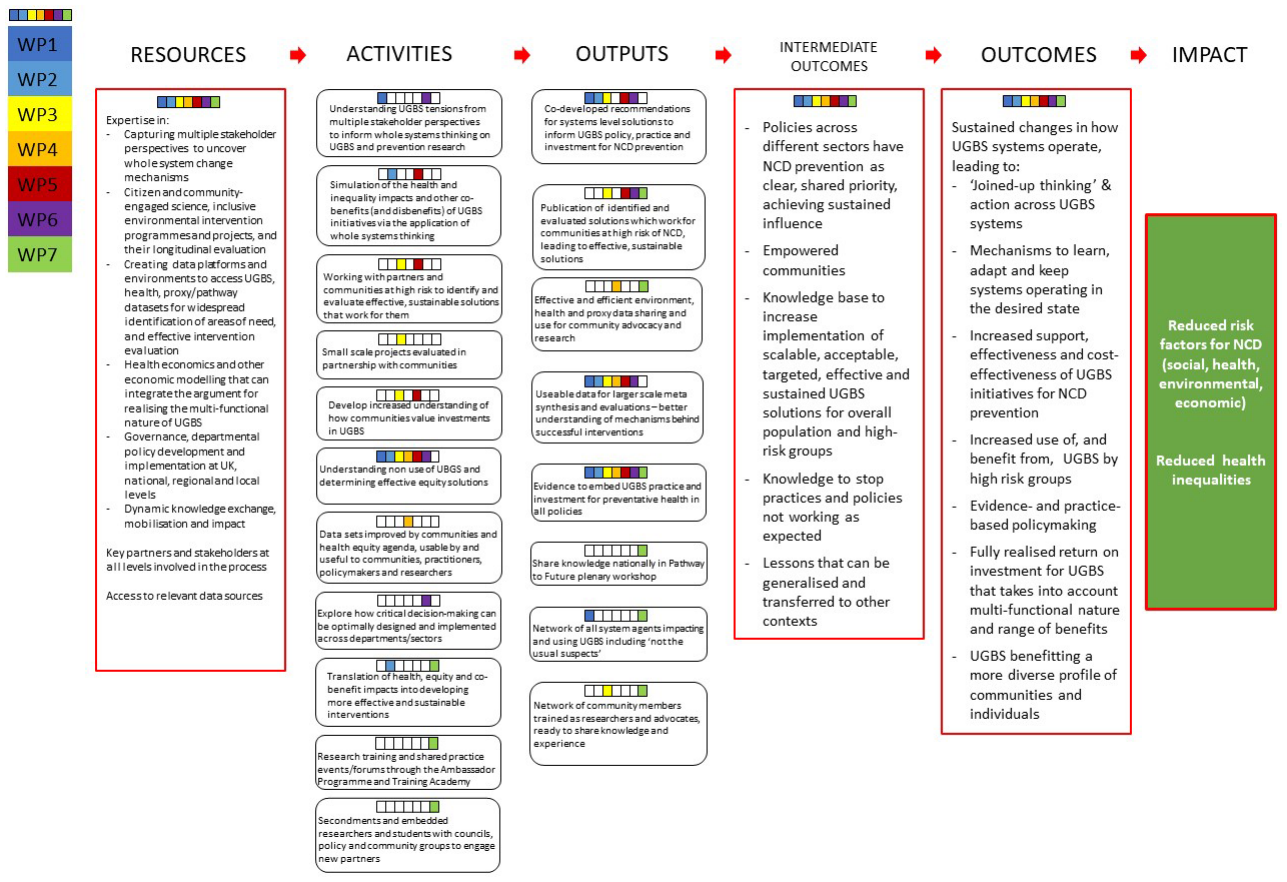
GroundsWell Logic Model.

Figure 1 details the Logic Model underpinning our Consortium.

## GroundsWell work packages

### WP1: Development of a system-oriented conceptual framework for shared understanding and transformations of UGBS

***Aim:*** Co-develop a systems-oriented conceptual framework that integrates multiple, layered, interconnected pieces of evidence, building a shared understanding of the mechanisms linking UGBS, NCD and inequalities that informs systems transformations of UGBS.

***Objectives:*** 1) Co-develop a conceptual framework underpinning GroundsWell that dynamically evolves as populated by our data and learnings; 2) Co-develop and implement a framework for system-based knowledge mobilisation and synthesis.

***Methods:*** A conceptual framework will be developed by combining; a) perspectives of multiple stakeholders collected using Soft Systems methods, Viable Systems Modelling and participatory Group Model Building methods; and, b) a systematic review of mechanistic pathways ^30^ for exposure-outcome relationships between UGBS factors, inequalities and NCD ^30, 31^. This combined approach should allow us to go beyond existing frameworks ^32^ by integrating scientific and practice-informed evidence, systematically analysing underlying values, beliefs and mechanisms, and explicitly considering feedback loops. Group Model Building workshops will be conducted with 15–20 stakeholders in each pioneer city (local communities, industry, researchers, national- and local-level government agencies) using the Community-Based System Dynamics approach ^33^. Building on and expanding from the findings of existing reviews ^32, 34– 37^, we will adapt the methodology developed by Lewis *et al*. ^30^ for systematic review of exposure-outcome mechanisms, which differ from traditional systematic reviews by identifying potential mechanisms underlying observed associations between an exposure and an outcome and systematically reviewing mechanistic pathways of interest (i.e. pathways between UGBS factors and NCD, informed by the causal-loop diagram). A conceptual framework will be created combining the stakeholders’ perspectives and systematic reviews, adapting a Contribution Analysis approach ^38^. We will challenge edges, chain of edges or subgraphs of the causal loop diagram initially using the evidence from the review detailed above, but as the project progresses, we will incorporate our own findings and lessons to continually update the conceptual framework, creating a dynamic knowledge mobilisation process within and beyond GroundsWell.

### WP2: Informing and simulating system-based UGBS transformations using agent-based modelling

***Aim:*** Co-develop and implement Agent-based Models (ABMs) that guide, facilitate, and contribute to: 1) hypothesis generation and data collation; 2) synthesise learning, and use of multiple layers of evidence to inform future UGBS interventions (such as programmes to improve UGBS quality or to increase the quantity of UGBS).

***Objectives:*** 1) Co-develop and implement a stylised (i.e. abstract “toy model”) ABM, complementing our conceptual framework, to generate hypotheses and inform data collation; 2) Co-develop and implement a virtual platform for simulating a portfolio of future UGBS interventions and their potential impact on a variety of city-wide NCD, inequalities, and co-benefit indicators.

***Methods:*** A stylised ABM, informed by the conceptual framework developed in WP 1, will be built to serve as a tool to assist the team to gain a deeper insight into the intricate dynamics in the system and their implications; generate new theoretical propositions and hypotheses; and inform data collation for GroundsWell ^39^. We will build on the stylised ABM to develop a data-informed ABM in which a portfolio of potential future UGBS interventions (defined in consultation with stakeholders, including, for example, users and non-users of UGBS, local authorities, Government, civil society) ^40^ and their impacts on NCD, inequalities and co-benefits can be experimented *in silico* to inform policy action. In line with ecological models ^41^ and the multi-level theory of population health ^42^, both models will account for social norms and shared values, urban and landscape design and inequalities in access to and quality of UGBS, and individual attributes (including opinions/beliefs and behavioural decisions regarding UGBS) that affect the decision-making. The data-informed ABM will draw strongly from WPs 3-6 results, secondary data analysis, and the health inequalities cross-cutting theme.

### WP3: Community innovation, co-production and citizen science for UGBS interventions

***Aims:*** To co-develop and evaluate processes for: 1) meaningful partnership working, and 2) citizen science approaches that lead to health enhancing and evidence based UGBS interventions.

***Objectives:*** 1) Co-develop and evaluate processes to enable communities at most risk from NCD to have meaningful involvement in decision-making processes about enhancing the health benefits of their local UGBS; 2) Co-develop and evaluate small scale (but situated within the complex system) UGBS projects to test these processes; 3) Use citizen-science approaches and tools to generate real-time ‘signals’ in the system and data on NCD outcomes. We focus on communities in the three cities that are at high risk of NCD and often excluded groups such as refugees, people with mental health issues.

***Case study sites:*** 1. *Existing* projects in the 3 cities including further improvement of the Connswater Community Greenway (Belfast) and Dock Branch Park (Liverpool); outdoor mental health programmes delivered by CHANGES (Edinburgh); 2. *New* projects developed as part of the Consortium. The exact interventions will be co-designed with our communities to address their needs and aspirations, working in collaboration with our stakeholders. 3. *Future* projects which are already planned or in development (e.g. greening of vacant, derelict or under-used land where micro-level interventions can be tested). GroundsWell will respond to the evidence suggesting the need for infrastructure improvements alongside social/educational/promotional programming ^4, 26, 43^, and evaluate these different types of intervention that fall within the single complex system around UGBS.

***Methods:*** We will develop a model for partnership working and specific UGBS which need improvement. We will develop and test models for integrating systems science and community engaged research drawing on the research in childhood obesity ^23^. We will then work with the case study hub using the 6SQuID framework ^44^ to: i) understand the problem (e.g. contested space, safety concerns, lack of use by certain population groups); identify the modifiable factors; create a theory of change and theory of action (intervention); test the intervention and undertake small scale evaluation; continually monitor, adjust and refine to achieve optimal conditions and outcomes. Evaluation designs will be equity sensitive and contingent on the phase of interventions (e.g. new, existing or planned) but may include realist, pre-post controlled and natural experiments. Individual outcomes include: UGBS use, physical activity, wellbeing, place belonging, environmental mastery, personal growth, relations with others and purpose in life. We will make use of advances in technology and citizen science to collect real-time, standardised data on NCD risk factors. Citizen involvement facilitates aggregation and synthesis of standardised data from small projects in diverse populations. Citizen science-oriented data collection methods will include Our Outdoors App ^45^, other outcome data through a participatory Delphi approach and interviews.

### WP4: Developing a dynamic data repository for pioneer cities to evidence system-wide benefits of UGBS interventions

***Aim:*** Generate a repository of well-curated, policy relevant, research-ready UGBS and linked NCD data for our pioneer cities, to inform the co-design and evaluation of large-scale UGBS interventions.

***Objectives:*** 1) Extract, connect and harmonise existing diverse UGBS and NCD data sources; 2) Evaluate the impact of meso- and macro- scale UGBS interventions on NCD, health inequalities, wider co-benefits; 3) Co-develop a platform to share data on current UGBS locations, needs and benefits; 4) Collation and mobilisation of GroundsWell data (with WP1 and 7).

***Methods:*** A repository accessible by stakeholders will be built to contain UGBS data, and extract UGBS features to demonstrate current city-wide UGBS locations and local needs by health inequalities. We build on international work theorising the relationship between people, nature and health, and more practically, augment UGBS access measures for UK regions ^32, 34– 37, 46, 47^, by bringing the ‘usual’ data together (vector, satellite, administrative records) to create small area UGBS indicators ( Place-Based Longitudinal Data Resource) ^48, 49^. We will advance previous work to create data suitable for household-individual level linkages to build longitudinal cohorts within our data safe havens ^50^. Linkage systems capable of ‘tracking’ anonymised *individuals* most in need, will be created to ensure that we do not miss effects for at-risk populations within a predominantly healthy population ^51^. Embedded researchers, acting as knowledge brokers, will promote to stakeholders the importance of spatio-temporal environmental data for linkage ^52^. We will use neural networks to extract perceptions of parks from social media data ^53, 54^ and use street view imagery ^55, 56^ to classify UGBS quantities and qualities important to our stakeholders ^57^. GroundsWell will harmonise these data, allowing analyses across and between our cities. The data team will evaluate large scale interventions in our cities to benefit the population(s) most at risk of developing NCD.

### WP5: Economic evaluation innovation for capturing UGBS system-wide benefits

***Aim:*** To examine the whole system “societal” economic case for UGBS interventions.

***Objectives:*** 1) Co-explore the role of the social economy and community assets, and assess their significance, for development and implementation of UGBS interventions; 2) Investigate people’s preferences and monetary values for UGBS, particularly exploring UGBS use for those at high risk of NCD; 3) Evaluate the social return on investment (SROI) in UGBS capturing system-wide benefits.

***Methods:*** Distinct methods will be used with respect to each objective. For objective 1, we will explore how social enterprises, cooperatives and co-ownership models can capture, sustain and recycle the value of UGBS interventions using a desk review of global models of practice (n=20); an audit of social enterprises delivering UGBS in each pioneer city; a set of case studies (n=5 per city) reflecting the range of social enterprise responses; and an evaluation of analysis of leading social enterprise response in each city (n=3). For objective 2, a discrete choice experiment (DCE) will examine the value of UGBS and the relative importance of barriers and enablers for UGBS use for those at high risk of NCD. Piloting of the questionnaire and analysis of the pilot data will provide new priors for updating the experimental design and improve the efficiency of the design itself for sample size calculation ^58^, aiming for 800 participants in each pioneer city. The target population will be members of community groups from areas of high deprivation that will be contacted through our stakeholders. Heterogeneity in values will be examined using a range of econometric techniques including deprivation and NCD risk exposure. For objective 3, a SROI approach will be used to provide a fuller picture of system-wide UGBS health and co-benefits, and their distribution across various communities. This will build on our SROI of UGBS in Belfast and Edinburgh ^59, 60^ and the findings from WP1 to include the values that matter for stakeholders, for example, health, wellbeing and NCD risk factors, and public health and planetary health co-benefits such as tourism, biodiversity, crime, and employment, and extend it to include ‘green issues’ to consider the ‘business case’ for UGBS providing sustainable food, housing and energy.

### WP6: Political and decision-making contexts of UGBS for health actions and co-benefits

***Aim:*** Co-explore the decision-making arrangements and contexts of UGBS policies for health, co-benefits and reducing inequalities; identify pragmatic, systems-wide actions to improve policy and decision-making to promote equity and sustainability, and address power disparities.

***Objectives:*** 1) Work with decision-makers from across the nested systems to understand how UGBS for health and co-benefits are conceptualised and implemented, and where responsibility, accountability and agency are considered to lie; 2) Identify what works in improving co-beneficial policy and decision-making within a complex adaptive system and in enabling communities to play an active, informed and meaningful role in decision-making.

***Methods:*** Drawing on the conceptual framework in WP1, and complemented with systems mapping and network analysis ^61^, we will analyse the nested (horizontal (e.g. inter-departmental) and vertical (e.g. national-local)) policy and decision-making landscape and actors (e.g. political, 3rd sector, funding bodies, private). Documentary content analysis (e.g. legislation/policy) and other qualitative methods (e.g. interviews, focus groups) will explore the explicit and implicit motivations and drivers, conceptions of responsibility, agency and accountability held by key stakeholders.

Second, we will evaluate what systems-level UGBS policy and decision-making actions/approaches for preventative health are effective, for whom, under what circumstances, and how. We will work with decision-makers from across the systems to co-identify and prioritise promising policy and decision-making approaches, architectures, or levers within contexts linked to each pioneer city at macro-, meso- and micro-levels. Using a mixed method realist evaluation approach, we will explore what works to enhance UGBS decision-making for health in the selected cases ( *n* determined by scales of options), focusing on core governance factors derived from earlier WP6 activities including: statutory tools and levers; institutional robustness and flexibility to change and adaptive capacity; agency and accountability; co-benefits and cross-sectoral collaboration; funding and financing; and role of data, evidence, valuation and tools.

Third, we will critically explore the meaningful involvement of communities in (political and institutional) decisions. We will use qualitative comparative analysis (QCA) to conduct structured analysis across approximately 15 cases to identify the necessary conditions or intervention characteristics for effective involvement of communities in decision-making. We will work with pioneer city stakeholders to prioritise and develop key actions, processes, or tools, with the potential to support communities to participate in UGBS decision-making within the system in ways that are meaningful to, and driven by, them and which acknowledge histories of UGBS neglect or exclusion amongst specific groups.

### WP7: Embedding and evaluating impact

***Aim:*** To co-develop, implement, monitor and evaluate a stakeholder-informed impact strategy that creates and evaluates impact within the 5 years of funding and beyond.

***Objectives:*** 1) Understand where impact should and could be demonstrated, informed by stakeholders needs; 2) Co-develop a strategy with knowledge brokers and measures of performance to ensure capacity and expertise across stakeholders (in community, policy, practice, research) to deliver impact; 3) Embed measures of impact in monitoring and evaluation plans.

***Methods:*** We will undertake a survey of research users to evaluate the usability, utility and functionality of our outputs. A survey of key researchers working in the field of NCD prevention and UGBS will identify effective strategies for knowledge exchange and impact to local, national and international audiences ^62^. We will hold a 1-day workshop with participants of the GMB exercise in WP1, with emphasis on: i) synthesising and interpreting the evidence from each WP; ii) planning the orchestration of ongoing and planned multisectoral UGBS actions; and iii) agreeing best ways to sustain collaborations beyond the lifespan of the Consortium funding. Principles and stages of adaptive policy ^49^ and structured decision-making ^51^ will inform the workshop. A citizen jury ^63^ in each city (n=12–15 per Jury) will be held with people from across the life course and from disadvantaged communities, to ‘sense-check’ possible UGBS-based NCD prevention approaches and policies identified in the workshop. We will: i) hold a Pathway to the Future plenary workshop involving multisectoral stakeholders and communities and ii) engage with other UK towns and cities through the WHO Healthy Cities and UN Child Friendly Cities Networks, to help us interpret how our findings might apply to other contexts, informed by guidance on transportability ^52^ and transferability ^53^ and our understanding of the complex systems affecting NCD prevention ^54^.

### Cross-cutting theme: Health inequalities

There is a critical opportunity to ensure that improvements to the quality and characteristics of UGBS support and promote use – addressing socio-economic inequalities as well as quantity/proximity of UGBS – and are also more amenable to intervention than making more/closer spaces in the current economic climate. Those less likely to have access to and/or to use UGBS are more likely to have NCD; however, where less advantaged groups *do* access UGBS, the health benefits seem particularly marked. This suggests UGBS have genuine potential to contribute to reduce health inequalities. However, health inequalities are not routinely considered across UGBS research, policy and practice. Our objectives include: i) integrating and monitoring health inequalities considerations in all Consortium activities; ii) increasing competency, capability and awareness of health inequalities across staff, stakeholders and communities; iii) filling gaps in our understanding of how UGBS systems create or maintain inequality in access (iv) using the web resource For Equity ( https://forequity.uk/) we will work with our stakeholders to collect and integrate variables into administrative datasets that enable equity sensitive impact analyses; (v) developing case studies/ examples of how GroundsWell has integrated an intersectional health inequalities focus in its research activities, with a particular focus on quantitative methodologies; (vi) evaluating and reporting GroundsWell impact on health inequalities.

The theme will work across all WPs to: i) ensure, support and share consistent and comparable health inequalities thinking; ii) co-produce a theoretical model of non/low use of UGBS; iii) monitor community participation in GroundsWell; iv) embed a health inequalities focus in all data and analyses; and v) secure health inequalities considerations using the Health Inequalities Assessment Toolkit ^64^.

## Plans for co-production and knowledge transfer

GroundsWell uses four principles to ensure meaningful involvement of our stakeholders in all co-production activities: 1) power is agreed and acknowledged as being held jointly by all people involved; 2) there is active involvement in decisions that impact upon the project and evaluation of its success; 3) potential barriers to access and participation (including income, education, gender, ethnicity, age, disability, language, and caring responsibilities) are acknowledged and tackled; and 4) when appropriate and desired by the community, there is full and active involvement in implementation of the solutions ^21^.

## Opportunities and challenges

GroundsWell will build an evidence base for the public and planetary health co-benefits of UGBS in our cities and presents a significant opportunity to raise the profile of UGBS and the role they have in addressing critical contemporary health, social, economic and environmental challenges. Through explicitly acknowledging the complex systems linking UGBS, NCD and environment/health inequalities, the programme of work will deliver novel understanding beyond the constraints of single risk-factor epidemiology or traditional intervention evaluations. The consortium will have the capacity to co-produce evidence with immediate real-world value and implications for action to obtain human health benefits sustainably, as we foster the regeneration of the natural environments themselves in the process.

There are challenges when bringing together academics and stakeholders from diverse disciplines and perspectives. We anticipate communication and epistemological challenges due to differences in language and culture across disciplines but working together to create an interdisciplinary team is needed to bring fresh ideas. We will bridge the siloes of health and environment that are currently seen as mutually exclusive in many organisations. We have a dedicated team who will help build a ‘Team Science’ ethos ^62^ and help create a shared language and identity, to minimise these issues.

A significant challenge that is often overlooked when seeking 'transformation' but are heavily relying on co-production, co-design, etc. with people who are strongly embedded in current systems. There is a challenge in supporting stakeholders (including researchers) to think differently. We generally think about what is 'probable' (i.e. likely to happen) and it can often be a struggle to think about what might be plausible (i.e. could happen). We have embedded participatory tools, activities and techniques to support stakeholders to expand their thinking to consider what is 'possible' (i.e. might happen) to stretch our thinking.

We expect challenges in accessing routinely collected health data and combining these with environment data across time and space, at scales suitable to evidence what works and for whom to reduce NCD over long periods of time. The lessons and findings from the multiple work packages, each addressing particular aspects of UGBS, will provide rich material to form a holistic view to inform systems transformations of UGBS; however, skilfully handling the numerosity and variety of pieces of evidence, obtained across multiple levels, will be critical to build a coherently integrated systems-oriented framework.

We anticipate challenges in evaluating interventions and generating evidence regarding NCD prevention and reduction in health inequalities which are hard to measure. Extracting environmental influences on health can be difficult when they have smaller impacts than individual and biological drivers that local communities may be more concerned about. However, small effects are often meaningful, particularly when they benefit a large fraction of the population, and UGBS are potentially easier to modify than biomedical issues. From some parts of the system there may be a lack of trust in the evidence, and we will create robust study designs to overcome anticipated scepticism. We envisage challenges in engaging seldom heard groups, whose views are particularly important for us to understand issues such as non-use and inequalities in access. We will also need to work with communities to build trust and support them to engage with, articulate and communicate the benefits of UGBS, contribute to the evidence base, and generate bottom-up system changes.

## Data Availability

No data are available with this article.
