# Supplementary material for: GroundsWell: Community-engaged and data-informed systems transformation of Urban Green and Blue Space for population health – a new initiative
Source: Wellcome Open Res. 2022 Sep 20;7:237. [Version 1] doi: 10.12688/wellcomeopenres.18175.1 (PMC9971655; doi:10.12688/wellcomeopenres.18175.1)
Supplement: Peer review history

# Peer review: Reviewer response for version 1

**DOI:** 10.21956/wellcomeopenres.20149.r54784

**Published:** 2023-02-22

**Authors:** Simon Kingham

**Affiliations:** 1 GeoHealth Laboratory, University of Canterbury, Christchurch, New Zealand

This is an interesting programme and partnership which seeks to understand more about how to better plan, design, evaluate and manage urban green and blue spaces (UGBS) so that it benefits all communities, especially those with the worst health outcomes.

 The method is clearly and comprehensively laid out, and seems appropriate. The consortium is strong and looks appropriate for the project.

 I would like to see more detail on the engagement with stakeholders and end users. The project has potential to deliver “ *immediate real-world value*”, but the lack of detail on the “ *Plans for co-production and knowledge transfer*”, in other words ‘how will happen’, reduces the certainly of this happening. A little more detail would allay concerns and increase the chances or real impact.

 While I have no particular problem with the choice of the three ‘pioneer’ cities (which I assume are partly picked as the researchers are based or have prior connections there), I think some rationale/justification for their selection would be beneficial. For example, how are they representative of other types of cities, are they geographically, socially or demographically of particular interest? Do they have certain levels of UGBS? I also wonder whether one smaller town (perhaps under 50,000) might be interesting to study, although of course this may stretch the resource of the project.

Does the article adequately reference differing views and opinions?

Yes

Are all factual statements correct, and are statements and arguments made adequately supported by citations?

Yes

Is the Open Letter written in accessible language?

Yes

Where applicable, are recommendations and next steps explained clearly for others to follow?

Not applicable

Is the rationale for the Open Letter provided in sufficient detail?

Yes

Reviewer Expertise:

Urban environment and wellbeing, transport, policy

I confirm that I have read this submission and believe that I have an appropriate level of expertise to confirm that it is of an acceptable scientific standard.

# Peer review: Reviewer response for version 1

**DOI:** 10.21956/wellcomeopenres.20149.r52465

**Published:** 2022-11-02

**Authors:** Rosemary R. C. McEachan

**Affiliations:** 1 Bradford Institute for Health Research, Bradford, UK

Thank you for the opportunity to review the plans for the ambitious GroundsWell consortium. The GroundsWell project, with its focus on whole systems approaches, aims to explore who we can maximise the potential benefits of green and blue space for population health. 

 Key strengths of the consortium include the multi-disciplinary approach to methods, integrating citizen science, modelling, quantitative and qualitative approaches, and the focus on co-production and co-design which will result in impactful activities. 

 I have a few minor suggestions to which I think will aid flow and understanding of the manuscript:

- It would be useful to have a setting section early in the methods. Throughout the manuscript you talk about 'three pioneer' cities, and I think it would help the reader to understand a bit more about where these cities are and their characteristics.
- Co-production and co-design is mentioned throughout. I think it would be useful to outline your approach to co-production and co-design earlier in the manuscript, before you outline the work-packages. It would be good to understand *who *the stakeholders are and what methods you plan to use to engage with each of them. For example how you might engage with communities might be very different from policy makers. A bit more about how you plan to manage the co-production and co-design for the different work-packages, and how you will ensure community voices are heard would be useful.
- Page 7 - WP5, methods, typo midway down this paragraph - 'priors' should be 'priorities?'
- Please could you check the first sentence of the second paragraph on page 9 (e.g. A significant challenge....) - is there a word missing?

Does the article adequately reference differing views and opinions?

Yes

Are all factual statements correct, and are statements and arguments made adequately supported by citations?

Yes

Is the Open Letter written in accessible language?

Yes

Where applicable, are recommendations and next steps explained clearly for others to follow?

Not applicable

Is the rationale for the Open Letter provided in sufficient detail?

Yes

Reviewer Expertise:

Green and blue spaces, environmental determinants of health, intervention studies, cohort studies, co-production

I confirm that I have read this submission and believe that I have an appropriate level of expertise to confirm that it is of an acceptable scientific standard.
